# Seed-specific suppression of ADP-glucose pyrophosphorylase in *Camelina sativa* increases seed size and weight

**DOI:** 10.1186/s13068-018-1334-2

**Published:** 2018-12-18

**Authors:** GunNam Na, Niranjan Aryal, Abdelhak Fatihi, Jinling Kang, Chaofu Lu

**Affiliations:** 1 Department of Plant Sciences and Plant Pathology, Montana State University, Bozeman, MT 59717-3150 USA; 2 Present Address: IJPB, INRA, AgroParisTech, CNRS, Université Paris-Saclay, RD10, 78026 Versailles Cedex, France

**Keywords:** *Camelina sativa*, RNAi, Seed size, Seedling establishment, ADP-glucose pyrophosphorylase, Starch

## Abstract

**Background:**

Camelina (*Camelina sativa* L.) is a promising oilseed crop that may provide sustainable feedstock for biofuel production. One of the major drawbacks of Camelina is its smaller seeds compared to other major oil crops such as canola, which limit oil yield and may also pose challenges in successful seedling establishment, especially in dryland cultivation. Previous studies indicate that seed development may be under metabolic control. In oilseeds, starch only accumulates temporarily during seed development but is almost absent in mature seeds. In this study, we explored the effect of altering seed carbohydrate metabolism on Camelina seed size through down-regulating ADP-glucose pyrophosphorylase (AGPase), a major enzyme in starch biosynthesis.

**Results:**

An RNAi construct comprising sequences of the Camelina small subunit of an AGPase (*CsAPS*) was expressed in Camelina cultivar Suneson under a seed-specific promoter. The RNAi suppression reduced AGPase activities which concurred with moderately decreased starch accumulation during seed development. Transcripts of genes examined that are involved in storage products were not affected, but contents of sugars and water were increased in developing seeds. The transgenic seeds were larger than wild-type plants due to increased cell sizes in seed coat and embryos, and mature seeds contained similar oil but more protein contents. The larger seeds showed advantages on seedling emergence from deep soils.

**Conclusions:**

Changing starch and sugar metabolism during seed development may increase the size and mass of seeds without affecting their final oil content in Camelina. Increased seed size may improve seedling establishment in the field and increase seed yield.

**Electronic supplementary material:**

The online version of this article (10.1186/s13068-018-1334-2) contains supplementary material, which is available to authorized users.

## Background

Camelina (*Camelina sativa* L. Crantz) is a promising oilseed crop that may provide sustainable feedstock for biofuel production due to its ability to adapt to a wide range of environments and relatively low input requirements [1]. The cruciferous Camelina plant is also an excellent rotation crop to improve the sustainability of the cereal-based cropping systems [2]. However, as a recently reemerged crop, several agronomic traits need to be improved to make the production of Camelina economically viable. One of the most important breeding objectives is to increase seed size. Camelina small seed size (~ 1.5 mm × 0.8 mm, or 1 mg/seed) [3] may hamper its incorporation into modern agriculture that uses large farm equipment. Also the high risk of poor seedling establishment of current cultivars [4, 5] may be associated with the limited energy reserves (e.g., oil) packaged in a small seed, as was observed in Arabidopsis [6] and crop plants such as cotton and Indian mustard [7, 8]. Shallow planting (< 10–20 mm) somewhat improves seedling emergence but growers have difficulty planting this depth with their grain-planting equipment. In addition, periods of no rainfall after planting that dry out the soil surface also may prevent germination or kill the germinated seedlings. Increasing Camelina seed size and oil content would be of great value for boosting harvestable oil yield and for rapid field emergence and seedling establishment, particularly under less favorable growing conditions due to larger primary roots and hypocotyls.

Seed development of an angiosperm is initiated by the process of double fertilization followed by the formation of embryo and endosperm, which are enveloped by seed coat derived from maternal integuments. The rapid proliferation of the endosperm and growth of integument form a large embryo sac that strongly influences the final seed size of Arabidopsis and crop species such as soybean and canola [9, 10]. Genetic studies have identified several signaling pathways, including the Polycomb group proteins and their targets, the APETALA 2 (AP2) and MADS-box transcription factors, and the IKU pathway that regulate endosperm development, as well as a number of transcription factors that control the proliferation and expansion of seed coat [11, 12]. Seed development is accompanied by metabolic activities for the synthesis and accumulation of storage products including oil, protein and carbohydrates [13]. Therefore, besides genetic controls, the final size of a seed may also be influenced by metabolic activities [14].

Camelina seeds store oil and protein as major carbon and nitrogen reserves, respectively [15]. Like other Brassicaceae family plants such as *Arabidopsis thaliana* and *Brassica napus*, starch only accumulates during early embryo development but largely diminishes when seeds mature [16, 17]. Studies suggest that starch does not serve as a carbon storage sink in these plants, but the turnover of starch during oilseed development might be functionally linked to cell division and differentiation and also affects seed maturation processes [16]. Similar to the pathways in leaf tissue plastids, starch synthesis in embryos uses imported photoassimilates, mainly sucrose, as the carbon source. Sucrose is hydrolyzed into hexoses (glucose and fructose) by sucrose synthase or invertase to provide carbon skeletons and energy for seed growth and development. These enzymes are important to maintain metabolite homeostasis and carbon partitioning that may affect the process of seed maturation and consequently the pattern of storage compounds or seed size [18, 19]. Starch synthesis is initiated by the activation of glucose by converting Glc1P to ADP-Glc through an ADP-glucose pyrophosphorylase (AGPase) [16]. Reduction of AGPase activity led to an inhibition of starch synthesis in oilseed rape and delayed oil synthesis, although the final seed oil content was not affected [20]. Similarly, antisense suppression of AGPase in pea (*Vicia narbonensis*) reduced starch synthesis, and the alteration of carbon metabolism induced pleiotropic effects on water and nitrogen content, and a prolonged seed-filling period [21]. In this study, we investigated starch accumulation in Camelina seed and its effects on seed development and storage products by generating AGPase RNAi plants using a seed-specific promoter. The RNAi seeds accumulated moderately reduced starch corresponding to lower AGPase activities during seed maturation, but the oil content and fatty acid composition did not change in mature seeds. Interestingly, the seed size and seed mass were significantly increased. The enlarged seeds had enhanced seedling emergence when planted in deep soil in the greenhouse.

## Results

### Starch accumulation coincides AGPase gene expression during seed development

**Fig. 1:**
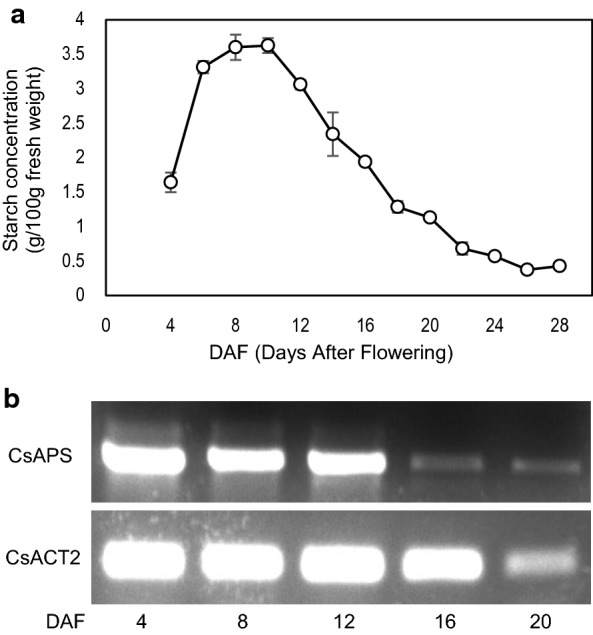
**a** Starch accumulation and **b** *CsAPS* gene expression during seed development in Camelina var. Suneson

To understand the starch accumulation pattern during Camelina seed development, we measured starch contents in developing seeds of a Camelina variety Suneson from 4 to 28 days after flowering (DAF). Results shown in Fig. 1a indicated that starch was already present in Camelina seeds at 4 DAF, and it increased rapidly during early developing stages to reach maximum levels during 8–10 DAF at about 3.6% of seed mass, then gradually declined so that only a small amount of starch (~ 0.4% of seed mass) was present in mature seeds. This pattern was similar to that in Arabidopsis seed [16]. As the AGPase is the major enzyme for starch biosynthesis, we monitored its expression by RT-PCR using RNA samples extracted from developing seeds of the same plants at 4, 8, 12, 16 and 20 DAF. Three genes encode AGPase small subunits (*CsAPS*) in Camelina (Csa11g074430, Csa18g014380 and Csa20g041200) which nucleotide sequences are nearly identical and show 93% identity to the Arabidopsis homolog (*APS1*, At5g48300) (Additional file 1: Fig. S1). Our results in Fig. 1b showed that high levels of *CsAPS* gene transcripts were detected during early stages of seed development (4–12 DAF), while a very low level of expression was detected after 16 DAF. This expression pattern coincided with starch accumulation.

### AGPase RNAi lines had decreased starch content

**Fig. 2:**
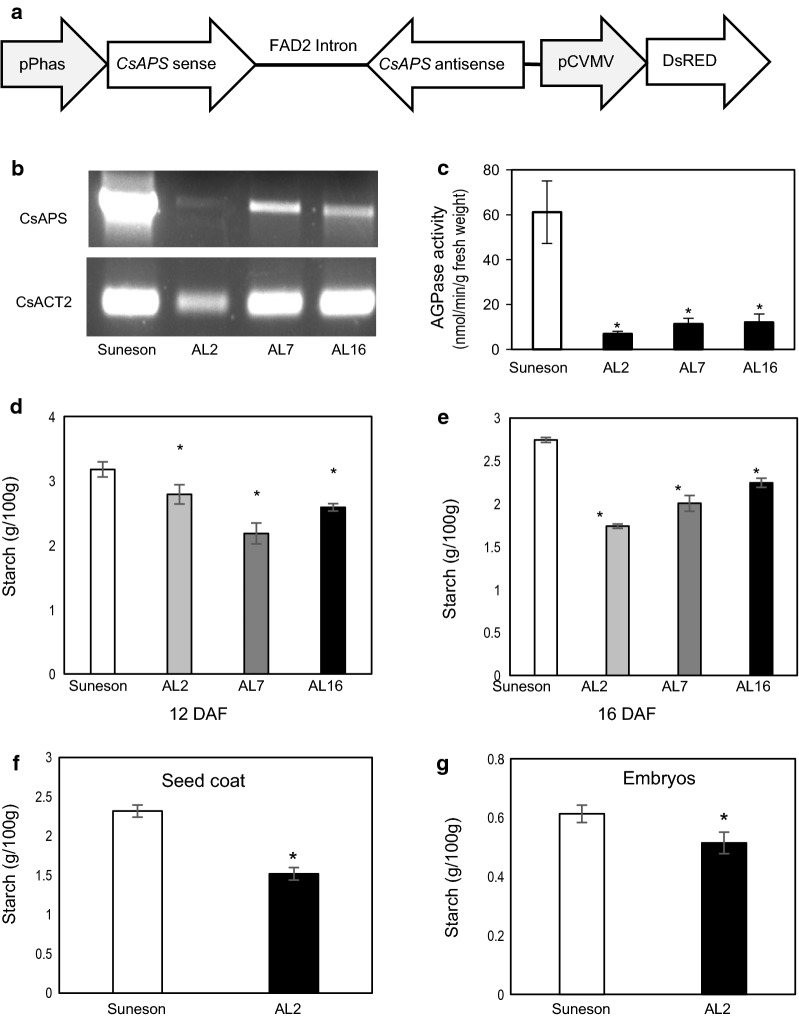
Suppression of AGPase in camelina seed reduced AGPase activity and starch content. **a** Schematic representation of the RNAi construct for the *CsAPS* genes. **b** The levels of *CsAPS* gene expression detected by semi-quantitative RT-PCR at 12 DAF. **c** Specific activity of AGPase in 12 DAF developing seeds. **d** Starch content in seeds at 12 DAF, and **e** 16 DAF. **f** Starch content in seed coat, and **g** embryos of 12DAF seeds. Starch contents were based on fresh weight of seeds or tissues. Data were obtained from three replicas of Suneson and three independent transgenic lines (AL2, AL7 and AL16). Significant changes from Suneson are marked with asterisks (P < 0.5)

An RNAi construct (Fig. 2a) targeting all three *CsAPS* genes was transformed into Suneson, and transgenic lines were selected using a DsRed selection marker [22]. Consequently, three homozygous plants were obtained that showed 3:1 segregation ratio for the DsRed expression in T2 and all seeds in T3 generations were red. These lines (namely AL2, AL7 and AL16) all showed decreased *CsAPS* gene expression, as indicated by RT-PCR at 12 DAF (Fig. 2b). The AGPase enzyme activities of the RNAi lines at this stage showed more than 80% decrease compared to the non-transgenic Suneson seeds (Fig. 2c). As a result, the starch content was also decreased at 12 and 16 DAF although it was less dramatic compared to the changes in gene expression and enzyme activity levels (Fig. 2d, e). Further examination determined that starch content in seed coat was almost four times that of embryos, and decreased in both seed coat and embryos, while more profound changes were found in seed coats (Fig. 2f, g).

**Fig. 3:**
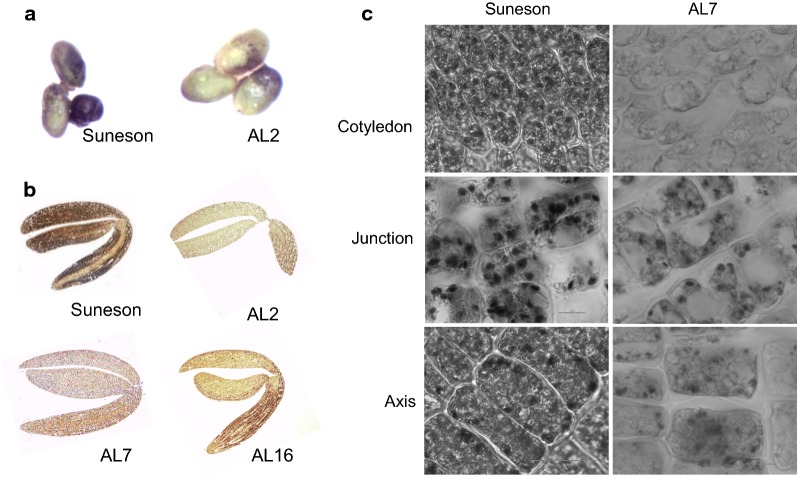
Camelina seed starch accumulation at 16 DAF shown by iodine staining. **a** Whole seeds of Suneson and AL2. **b** Embryos of Suneson and three transgenic lines. **c** Starch granules in Suneson and transgenic line AL7

To further confirm the RNAi effects, we used iodine staining to visualize the starch accumulation in Camelina developing seeds. Compared to Suneson, the transgenic lines showed decreased intensities of iodine staining in whole seeds including seed coats (Fig. 3a) and across the section of the embryos (Fig. 3b). Under the microscope, less and smaller starch granules were observed in different embryonic parts (cotyledon, axis and the junction between them) of the RNAi lines than those of Suneson. All these results indicated that RNAi suppression of the *CsAPS* genes in Camelina effectively reduced their expression levels, and thus caused significantly lower AGPase activities and reduced starch synthesis and accumulation in seed.

### AGPase suppression increased seed size and weight

To evaluate the effects of AGPase suppression on Camelina plant growth and seed production, we planted the RNAi lines along with Suneson in the field. Plant growth of the RNAi lines was nearly identical to Suneson so that all plants had similar height at maturation and produced similar amounts of seeds per plant (Additional file 1: Table S3). In mature seeds, the RNAi lines had similar levels of oil content compared to Suneson, and their fatty acid composition did not change significantly (Table 1 and Additional file 1: Fig. S3). However, the RNAi seeds contained about 8% more protein (Table 1). Interestingly, the seed mass (100-seed weight) of RNAi seeds was increased by 36–48% compared to Suneson. The RNAi seeds were also increased their sizes by 30–38% than Suneson (Table 1). These results were consistent over 3 years of growth in the greenhouse or field conditions (Additional file 1: Table S3).

**Table 1:** Comparison of seed traits between Suneson and the AGPase RNAi lines

Line	Weight (mg/100 seeds)	Size (mm^2^)	Oil (wt %)	Protein (mg/g)	Sugar (mg/g)
Suneson	101.1 ± 4.9^a^	1.3 ± 0.1^a^	32.4 ± 1.5^a^	182.6 ± 3.4^a^	56.3 ± 0.8^a^
AL2	136.4 ± 3.2^b^	1.7 ± 0.0^b^	33.3 ± 1.4^a^	197.5 ± 7.6^b^	55.6 ± 1.4^a^
AL7	149.1 ± 1.5^c^	1.8 ± 0.0^b^	35.3 ± 0.5^a^	198.4 ± 4.6^b^	55.8 ± 0.5^a^
AL16	147.5 ± 3.1^c^	1.8 ± 0.0^b^	34.4 ± 0.4^a^	196.8 ± 2.5^b^	59.4 ± 1.8^b^

Data represent averages of dry seeds from 10 individual plants harvested in 2016

^a, b, c^Different letters indicate statistical significances

**Fig. 4:**
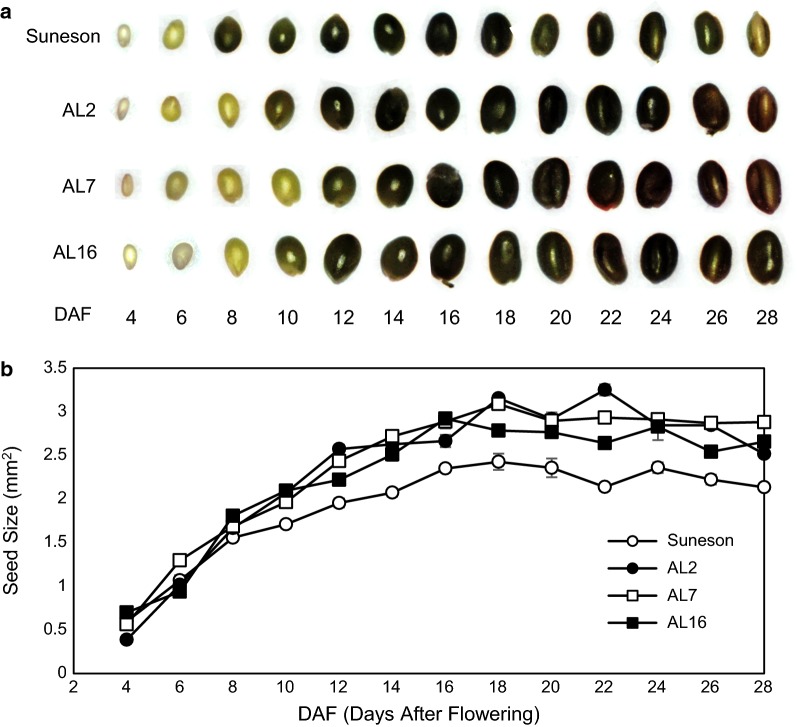
Comparison of seed growth during seed development between Suneson and RNAi lines. **a** Photos of seeds taken at different developmental stages; **b** differential seed size increases during seed development between Suneson and RNAi lines

We, therefore, measured the sizes of pods and seeds during seed development in greenhouse grown plants. Compared to Suneson, seed size of the RNAi lines did not show differences during early stages till 8 DAF. After that, the RNAi line seeds were significantly larger than Suneson and the differences remained in matured seeds (Fig. 4). The pod sizes were also slightly larger in the RNAi lines (Additional file 1: Fig. S2).

**Fig. 5:**
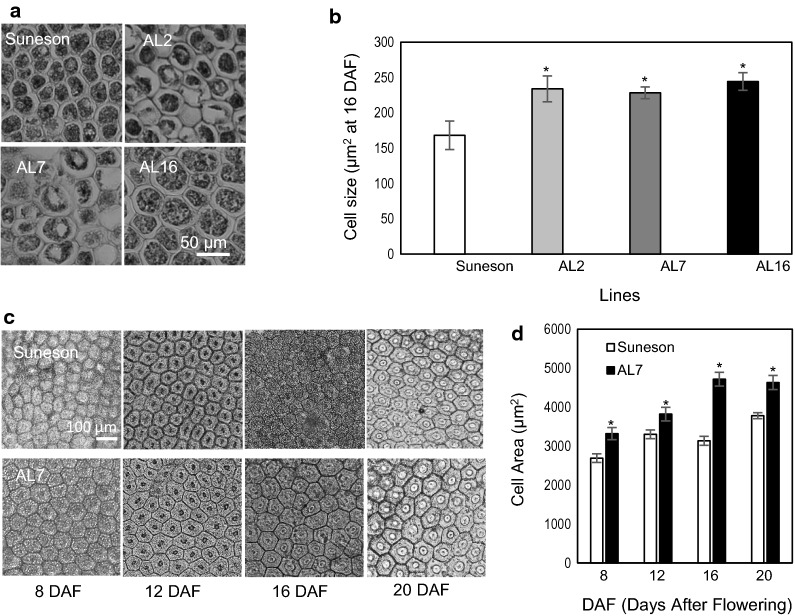
Microscopic analysis of cell size in seed coat and embryo. **a** Images showing cotyledon cells of Suneson and three independent transgenic lines at 16 DAF. **b** Chart showing differences in cotyledon cell sizes at 16DAF. **c** Microscopic analysis of seed coats from Suneson (top row) and transgenic line AL7 at 8, 12, 16 and 20 DAF. **d** Chart showing differences in cell size of seed coat. Values are the mean ± SE of four independent measurements. Significant changes from Suneson are marked with asterisks (P < 0.5)

The larger seed size suggested that the cell number per embryo or cell size might have increased in the RNAi lines. Since we used a seed storage protein (phaseolin) promoter that was active during maturation process [23], the increase of cell numbers was unlikely. To analyze possible changes in cell size, seeds collected at 8, 12, 16 and 20 DAF were imbibed and sectioned, and were examined under the microscope. Figure 5 shows that all three RNAi lines had increased cell size in seed coat and embryos. The average cell size of cotyledons in Suneson was about 170 μm^2^ at 16 DAF, while at the same developmental stage, the RNAi lines were about 230–245 μm^2^ (Fig. 5a, b). The cell sizes of seed coats also increased in the RNAi lines (Fig. 5c, d). These results indicated that the increased cell sizes in both seed coat and embryo caused increased final seed size in the AGPase RNAi lines.

### RNAi developing seeds had increased sugar and water contents

**Fig. 6:**
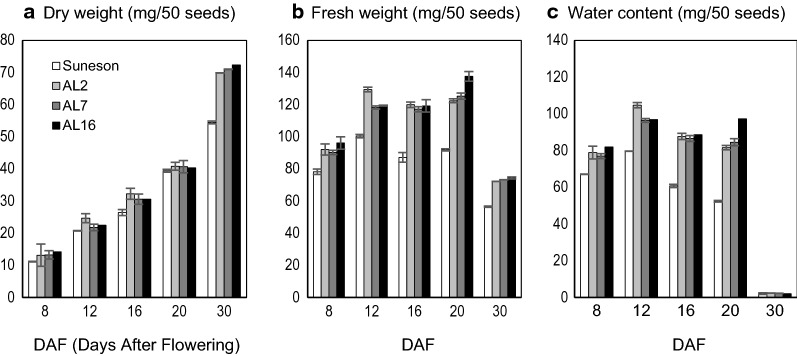
Developing seeds from AGPase RNAi transgenic lines contained elevated water content compared to control Suneson. **a** Dry seed weight, **b** fresh seed weight, and **c** water contents in developing seeds of Suneson and three independent AGPase RNAi transgenic lines at 8–30 DAF. Data represent means of three replica

We measured seed weight during seed development at 8, 12, 16 and 20 DAF. Seed dry weight gradually increased at nearly identical rates during this period for Suneson and RNAi lines (Fig. 6a); however, RNAi seeds gained more fresh weight during 12–20 DAF than Suneson (Fig. 6b). Water content, as calculated by the differences between fresh and dry weight, was increased in RNAi lines developing seeds. During active seed filling (12–20 DAF), the moisture content was evidently higher in RNAi lines than in Suneson, whose water content continuously decreased while in RNAi seeds it was steadily retained during the same period of seed development (Fig. 6c).

**Fig. 7:**
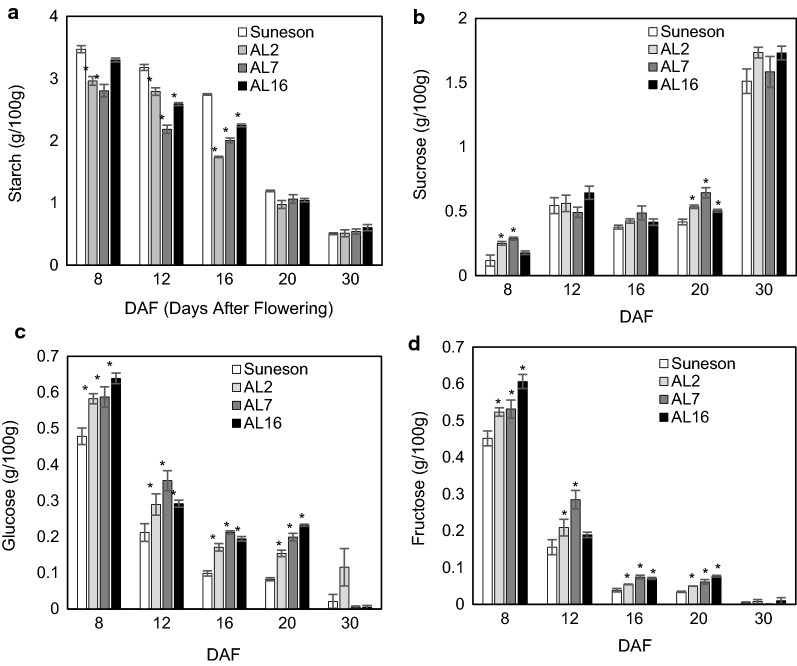
Effects of AGPase suppression on sugar contents in developing seeds. **a** Starch, **b** sucrose, **c** glucose, and **d** fructose contents in seeds of Suneson and three independent homologous AGPase RNAi transgenic lines during seed development. Significant changes from Suneson are marked with asterisks (P < 0.5)

We also measured the contents of sugars and starch in developing seeds. Starch is not a major storage compound in dry seeds. Compared to Suneson, the RNAi seeds contained similar levels of starch at 20–30 DAF, but significantly reduced amounts during early stages of seed development (8–16 DAF) (Figs. 2d, e, 7a). As shown in Fig. 7, sucrose is the major soluble sugar that is present in dry Camelina seeds (~ 1.5% of seed mass), with hexoses (glucose and fructose) being a negligible component. In Suneson, the sucrose level was low (~ 0.1%) during early stages of seed development (8 DAF), then it increased and remained at similar levels about 0.5% during 12–20 DAF. The RNAi lines had slightly increased sucrose throughout seed development and accumulated about ~ 1.8% in dry seeds. However, the hexoses especially glucose levels were significantly higher in all RNAi seeds during 8–20 DAF.

### Transcript levels of storage-associated genes were not altered

**Fig. 8:**
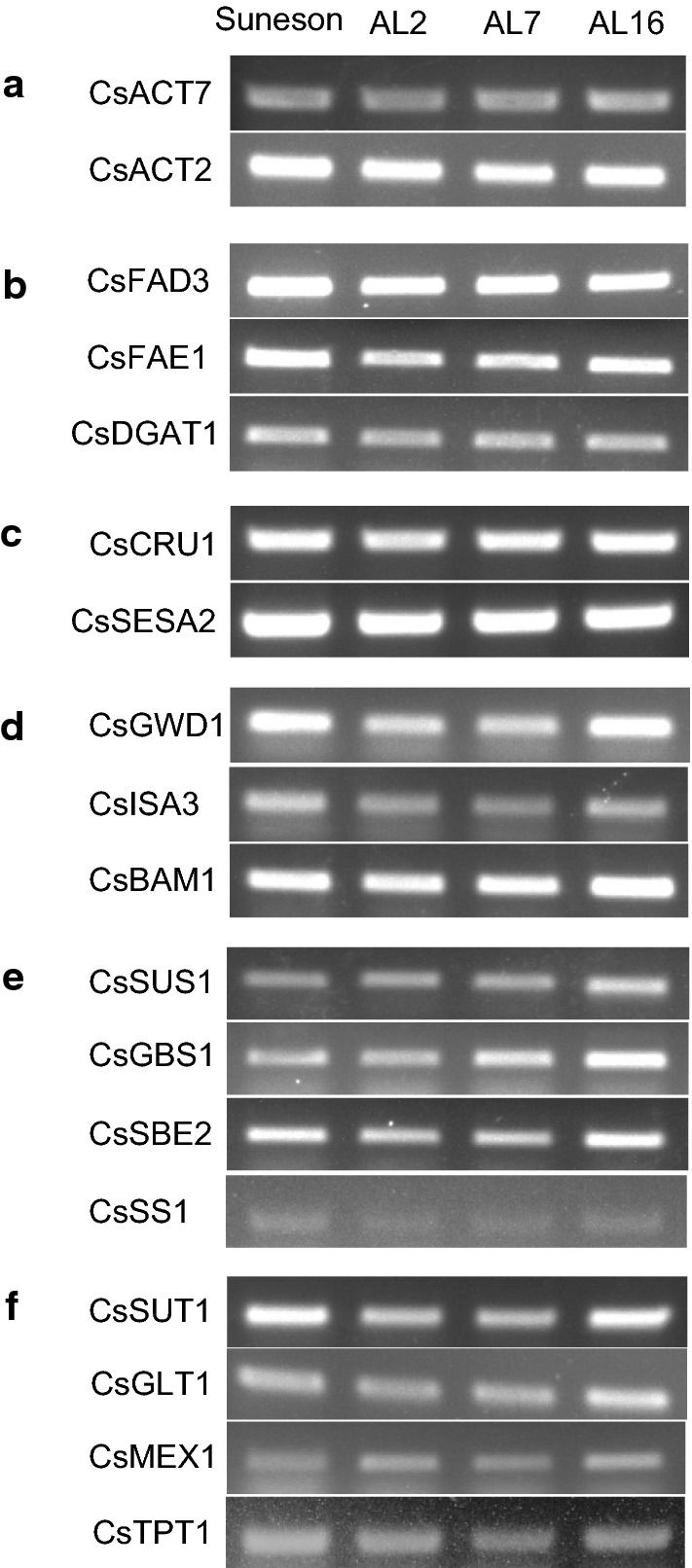
Transcript levels in Suneson and AGPase RNAi seeds at 12 DAF by semi-qRT-PCR. **a** Reference genes; **b** lipid biosynthesis genes; **c** storage protein genes; **d** starch degradation genes; **e** starch synthesis related genes, and **f** transporters. Gene names are ACT7 (Actin 7), ACT2 (Actin 2), FAD3 (Fatty acid desaturase 3), FAE1 (Fatty acid elongase1), DGAT1 (Diacylglycerol acyltransferase1), CRU1 (12S seed storage protein), SESA2 (Seed storage albumin2), GWD1 (Glucan water dikinase1), ISA3 (Iso amylase3), BAM1 (β-amylase1), SUS1 (Sucrose Synthase1), GBS1 (Granule-bound starch synthase1), SBE2 (Starch branching enzyme2), SS1 (Starch Synthase1), SUT1 (Sucrose transporter1), GLT1 (Glucose transporter1), MEX1 (Maltose transporter1), and TPT1 (Triose phosphate transporter1)

We analyzed the expression of genes related to metabolism of storage products. RNA samples were extracted from 12 DAF seeds and examined by semi-quantitative RT-PCR. We selected genes that are related to (1) lipid synthesis (*FAD3*, *FAE1* and *DGAT1*), (2) seed storage protein synthesis (*CRU1*, *SESA2*), (3) starch degradation (*GWD1*, *ISA3* and *BAM1*), (4) sucrose metabolism and starch synthesis (*SUS1*, *GBS1*, *SBE2*, *SS1*), and (5) sugar transport (*SUT1*, *GLT1*, *MEX1* and *TPT1*). Actin genes (*CsACT2* and *CsACT7*) were used as controls, whose expression showed similar levels in Suneson and AGPase RNAi lines (Fig. 8a). Results shown in Fig. 8 indicated that transcripts of these genes did not change significantly in RNAi lines compared to Suneson. We concluded that the suppression of AGPase gene expression did not cause alterations in other genes involved in metabolism of storage products.

### Larger seeds had enhanced seedling emergence

**Fig. 9:**
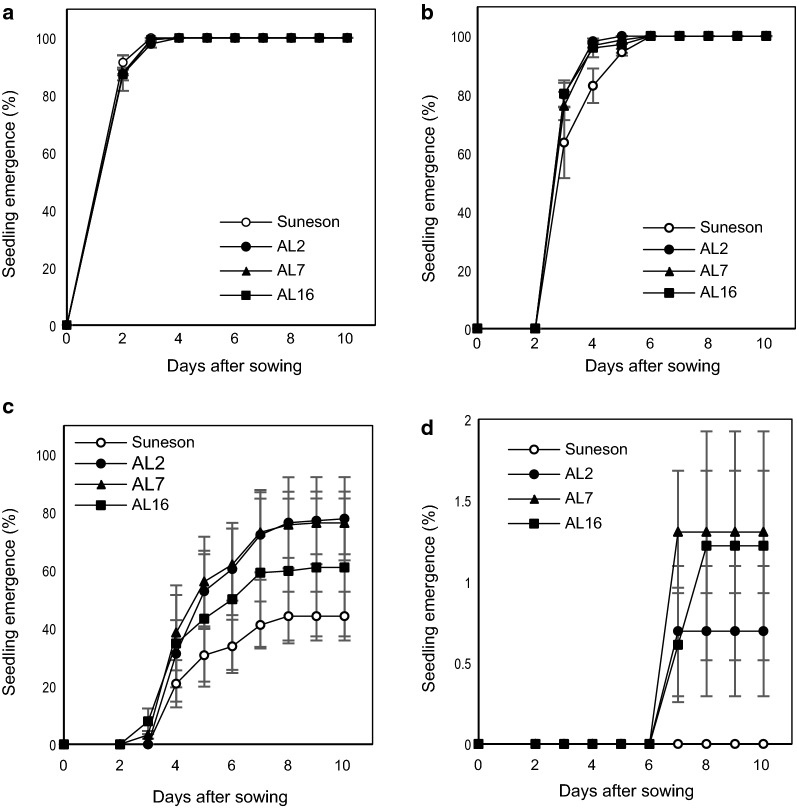
Seedling emergence test in soil at different sowing depth in the greenhouse. **a** 12.5 mm, **b** 25 mm, **c** 40 mm, **d** 50 mm

To examine the effect of AGPase suppression and starch reduction on seed vigor, we conducted seed germination experiments. When incubated on wet filter papers at room temperature, almost all seeds from Suneson and RNAi lines had germinated the following day (data not shown). Similarly, we did not observe differences between the RNAi and Suneson seeds in the soil in the greenhouse, where all lines showed seedling appearance from the soil surface in 2 days and nearly 100% had completed germination by 3 days after sowing (Fig. 9a). These results indicated that the AGPase suppression did not have negative effects on seed viability. We then tested the seedling emergence from soil by sowing seeds at different depths in the greenhouse (Additional file 1: Fig. S4). Compared to our usual seeding depth at about 0.5 inch (~ 12.5 mm), seedlings from seeds buried under 25 mm soil appeared 1 day later (starting from day 3), but seedlings from all the RNAi lines appeared faster than Suneson (Fig. 9b). This difference became more profound when seeds were sown at about 40 mm depth (Fig. 9c). At about 50 mm depth, we did not see seedling emerged from Suneson, while only a few from the RNAi lines (Fig. 9d). These results suggested that larger seeds caused by AGPase suppression might have conferred improved ability of seedling emergence from deep planted soils.

## Discussion

Seed size is an important life-history trait defining the fitness of a plant, and larger seeds had been generally favored during early cultivation and crop domestication [24]. Greater burial depth might not have been a general selection pressure during crop domestication [25], but studies indicated that larger seeds improved seedling survivorship [26] and generally performed better in the field [27]. Seed size and weight, which are often positively correlated to each other and seed yield, are therefore the targets of the most important traits for crop improvement [28]. Camelina has not been subject to intensive modern breeding though it is a crop with a long cultivation history [29]. Increasing seed size in Camelina would be much beneficial for higher yield, but also may enhance its agronomic performance in dryland cropping system by improving seedling establishment.

In this study, we demonstrated that disrupting starch biosynthesis in Camelina seed increased its size and mass. We provided evidence that seed-specific RNAi suppression of the AGPase small subunits *CsAPS* genes successfully reduced their transcription and the AGPase activity, thus resulting in significant albeit modest reduction of starch accumulation in developing Camelina seeds. This metabolic modification did not cause any significant penalty for storage products accumulation as the oil content and fatty acid composition did not change while protein content was slightly increased. Since we did not see differences in the amounts of seeds per plant, the larger and heavier seeds in the RNAi lines may be translated into higher yield. This aspect needs to be tested in larger scale in the field than our present study which only planted a limited number of plants per trial. Most significantly, the larger seeds enabled seeds to emerge from deep soils in our greenhouse experiments. This may allow for deeper planting in the field to improve seedling establishment in dryland Camelina production.

Seed size is a complex trait that is controlled by multiple genes in coordinated networks and may also be influenced by metabolic activities during seed development. To understand the mechanisms that may have caused increased seed size, we examined gene expression and seed growth. Transcripts of several genes examined that are involved in metabolism of seed storage products were not changed in the RNAi lines, suggesting that suppression of starch synthesis in Camelina did not affect expression of these genes. Although we could not rule out any possible changes in the seed transcriptome that include genes affecting seed size, we hypothesized that it was more likely the decreased starch synthesis that might alter the metabolic homeostasis and consequently cause the increase in seed size. Sucrose translocated from photosynthetic tissues provides the carbon source of lipid and starch synthesis in developing seeds [30, 31]. Sucrose is broken down to hexoses, whose metabolism provides for metabolic and energy needs so that their levels were relatively high during early stages (e.g., 8 DAF) of seed development. Similar levels of these soluble sugars were observed during seed filling periods, and in mature seeds only sucrose was noticeably present. Starch is a form of temporary carbon sink that only a small amount was present in mature seeds. These trends of metabolic dynamics in Camelina seed are similar in other oilseeds like Arabidopsis and rapeseed [16]. In AGPase suppressor lines, soluble sugars were higher than Suneson throughout the developmental stages (8–20 DAF). Interestingly, the water content was higher in the RNAi seeds during seed maturation. These observations suggest that the higher concentrations of osmotically active sugars in the RNAi developing seeds might have caused higher water uptake than Suneson. Regulation of turgor pressure plays a key role in plant cell growth. Seed development involves the coordinated growth of maternal seed coat tissues and the enclosed zygotic endosperm and embryo. In oilseed rape and Arabidopsis, it has been demonstrated that the accumulation of high concentrations of hexoses and other soluble metabolites may decrease the osmotic potential of cells in endosperm and other tissues to drive water uptake [32, 33]. Early seed expansion and growth arrest are associated with the rise and drop of endosperm-derived turgor pressure [33]. Our observations on increased water contents in the RNAi seeds were consistent with their enlarged cell sizes in seed coat and embryos in developing seeds. Earlier studies in peas also showed that altered starch metabolism in seed caused changes in seed shape and size [34, 35]. We, therefore, concluded that the metabolic changes in developing seeds due to lower AGPase activity and starch synthesis might explain the increased final seed size in engineered Camelina.

Interestingly, our results were different from a similar experiment previously conducted in *Brassica napus*. In that study [20], embryo-specific suppression of AGPase caused a delayed oil accumulation though the final oil content in mature rapeseed was near-normal; however, it did not lead to increased levels of soluble sugars and seed size. These different results might be mainly attributed to different promoters for driving AGPase RNAi expression. An embryo-specific thioesterase promoter was used in the rapeseed study, which resulted in decreased AGPase expression and starch synthesis restricted in the embryo. In our present study, AGPase RNAi inhibition driven by a strong seed-specific phaseolin promoter caused decreased starch in both embryo and seed coat, and more profoundly in the latter (Fig. 2f, g). Starch is found in embryos and seed coats of developing seeds of Arabidopsis [16] and Camelina (Fig. 2f, g), and seed coats contain nearly four times that of embryos around the peak periods of starch accumulation. The occurrence of high carbon flux toward starch in the testa coincides with the rapid cell division at the early stages of seed development [36]. The spatial and temporal patterns of starch accumulation in developing seed imply the roles of starch in cell division and differentiation besides its metabolic functions as a temporary carbon reserve [16]. Our results agree with the notion that seed coat development is a major factor determining seed size [10] and suggest that tissue-specific manipulation of gene networks and metabolic pathways may provide effective tools to alter seed coat development and increase seed size in Camelina.

## Conclusions

Seed-specific suppression of the Camelina ADP-glucose pyrophosphorylase small subunit (*CsAPS*) genes by RNAi decreased AGPase activity and starch synthesis. This transgenic interference altered sugar metabolism during seed development and consequently increased seed size and seed mass, while oil percentage content and fatty acid composition remained unchanged. The enlarged seeds germinated normally and showed enhanced seedling emergence from deep soil under greenhouse conditions. These results suggest that modifying carbohydrate metabolism during seed development may be an effective strategy to improve Camelina seed traits for biofuel production from this promising oilseed crop.

## Methods

### Plant material and growth conditions

A *Camelina sativa* cultivar “Suneson” was used for all experiments. Camelina was grown in 90 mm pots filled with soil and Sunshine Mix (Clinton, OK, USA) (1:1) in the greenhouse. To harvest pods and seeds at every 2 days after flowering, flowers were tagged using a thin slice of scotch tape around the pedicels. All samples were harvested in the middle of the light period (between 2 and 3 pm), put in liquid nitrogen and immediately stored at − 80 °C until further analysis. To test germination rate at different sowing depth, 60 seeds of wild type and three independent RNAi lines were planted 12.5 mm, 25 mm, 40 mm and 50 mm below the soil surface. Growth condition in the greenhouse was 20 °C day/16 °C night with natural light. Seeds were considered to have germinated when the cotyledons appeared, and seedling emergence was calculated as a percentage.

Plants were also grown in the field at the Arthur Post Agronomy Farm near Bozeman, Montana. Transgenic plants were planted in compliance with the release permits obtained from the Animal and Plant Health Inspection Service (APHIS) of the US Department of Agriculture. Seeds were sowed around May 5th and were harvested around August 15th each year (2014–2016).

### Construction of a binary vector and generation of transgenic plants

To generate a pGDP-AGPase RNAi plasmid, an AGPase small subunit (*CsAPS*) coding fragment sequence (586 bp) was amplified from a Camelina seed cDNA library [37]. The PCR product was cloned into a pGEM T-Easy vector (Promega) for sequencing. The sequences match completely with the gene Csa18g014380. Since this gene only differs from other two *CsAPS* homologs for 2 and 7 nucleotides in this region, we reasoned that the RNAi construct would be able to target all three *CsAPS* genes in Camelina for silencing. The PCR fragment was then placed under a seed-specific phaseolin promoter in sense and antisense orientations flanking an Arabidopsis *FAD2* intron to build an RNAi construct as described previously [37] (Fig. 2a). Primers (Additional file 1: Table S1) AGPS1-F and AGPS1-R with BamHI and NheI linkers, respectively, were used to amplify the sense fragment and AGP1AS-F and AGP1AS-R with XhoI and PstI linkers, respectively, were used to produce the antisense fragment. Primers FAD2 intron-F and FAD2 intron-R with NheI and PstI linkers, respectively, were used to amplify the *FAD2* intron fragment. The fragments were assembled as previously [37]. The pGDP-AGPase RNAi plasmid was introduced into *Agrobacterium tumefaciens* strain GV3101 by electroporation. Camelina plants were transformed using vacuum-mediated floral dipping as described previously [22]. Transformed plants were screened by detecting red fluorescence seeds. The T2 lines, showing a ~ 75% red fluorescence seeds (single insertion), were selected. T3 seeds collected from the individual T2 plants were again selected with red fluorescence, and only lines showing 100% red fluorescence were selected as homozygous.

### Measurement of seed size and weight and microscopic analysis

The size of dried seeds was determined by measuring their length and width using the Smartgrain software described previously [38]. The cell size of cotyledons was determined by measuring the area of embryonic cotyledon cells using the NIH ImageJ software (https://imagej.nih.gov/). The weight of 100 seeds was determined using a microbalance with four replications, and the average was calculated. To determine curves of fresh and dry weight accumulation, developing seeds of wild type and three independent AGPase RNAi lines were weighted and then seeds were dried to constant weight. The water content was calculated from the difference between fresh and dry weight. For microscopy of sections, developing seeds of wild type and RNAi lines were fixed in 3.7% formaldehyde in phosphate buffer at room temperature under vacuum for 4 h and rinsed with phosphate buffer three times. Fixed samples were dehydrated in an ethanol series using Tissue-Tek VIP6 Processor (Sakura Finetek USA, CA, USA) and were embedded into paraffin using a Tissue-Tek TEC embedding station (Sakura Finetek USA, CA, USA). Sections were cut at a thickness of 5 µm on a Leica ultra-microtome (Leica Microsystems, Milton Keynes, UK) and stained with Hematoxylin for 1 min or with iodine solution (KI 2% (w/v), I2 1% (w/v)) for 30 min and viewed with a Nikon Eclipse 800 microscope.

### Measurement of seed storage products

The oil content in seeds was measured using methods described previously [39]. For total protein measurement in seeds, the total protein extraction from seed samples was prepared using methods described previously [40]. The protein content was quantified using the bicinchoninic acid assay [41] with bovine serum albumin as the standard. For soluble sugar measurement in seeds, we used the procedure [42]. Ten microlitres of the extract was incubated with 1000 μL anthrone reagents [0.15% (w/v) anthrone, 72% (v/v) H_2_SO_4_, 28% (v/v) H_2_O] at 100 °C for 1 h. The absorbance was measured at 625 nm with glucose as the standard. Sucrose, glucose, and fructose were extracted by grinding 50 mg fresh weight of camelina developing seeds using methods described previously [43]. In brief, the powdered samples were extracted using extraction buffer (80% ethanol and 10 mM HEPES–KOH pH 7.4) two times at 70 °C for 2 h and at 65 °C for 24 h. Each extraction was centrifuged for 30 min at 10,000 rpm at room temperature, and each supernatant was collected and combined. Measurements were performed spectrophotometrically using the sucrose/d-glucose/d-fructose Megazyme Kit (Megazyme International Ireland Ltd, Wicklow, Ireland), according to the manufacturer’s description. The absorbance of the final mixture for each sample was measured at 340 nm. Starch was determined using a Starch Megazyme kit (Megazyme International Ireland Ltd, Wicklow, Ireland) as described previously [44]. The absorbance of the final mixture for each sample and d-glucose control was measured on a spectrophotometer at 510 nm against the reagent blank.

### Semi-quantitative RT-PCR

Developing Camelina seeds were harvested, immediately frozen in liquid nitrogen and stored at − 80 °C until further analysis. Total RNA was extracted from around 100 mg of developing seeds using mirVana miRNA isolation Kit (Life Tech, Carlsbad, CA, USA), and measured by NanoDrop (Nanodrop Technologies, Wilmington, DE, USA). First strand cDNA was synthesized from 10 µg total RNA using oligo-(dT)_23_ primers (Invitrogen) with the Superscript III reverse transcriptase (Invitrogen) according to the manufacturer’s instructions. One µL of cDNA product was used for the PCR. The primer pairs used for the PCR are listed in Additional file 1: Table S2. Primer sequences were designed to span intron regions to detect genomic DNA contamination when genomic sequence was available. The PCR products were amplified with the following conditions; 95 °C for 5 min, 18 to 26 cycles of 95 °C for 30 s, 58 °C for 30 s, 68 °C for 30 s and 68 °C for 10 min.

### AGPase activity in wild type and RNAi lines

Enzyme activity in the 12-day-old seeds was measured using Rosti et al. [45] method. Seeds were homogenized in 0.5 mL of extraction buffer containing 50 mM HEPES (pH 7.4), 2 mM MgCl_2_, 1 mM DTT. After centrifugation for 15 min at 10,000*g*, the supernatant was assayed for AGPase activity. Assays contained, in a volume of 0.2 mL, 100 mM HEPES (pH 7.6), 15 mM MgCl_2_, 0.025% (w/v) BSA, 0.5 units inorganic pyrophosphatase, 0.5 mM [U–^14^C] glucose 1-Phosphate, 1.5 mM ATP, 15 mM glycerate-3-phosphate (3-PGA), and 0.1 mL extract. Enzyme activity was determined by counting ^14^C from ADP-Glucose (labeled with ^14^C) which was the product of [U–^14^C] glucose 1-Phosphate and ATP. Specific activity was calculated by multiplying counts per minute with fletcher value (0.006) and dividing the product by 0.833 which is mg of sample counted for ^14^C in 10 min.

### Statistical analysis

Statistical analyses were performed using Microsoft Office Excel 2016. One-way analysis of variance was done to test the equality of three or more means. Student’s *t* tests were done to statistically compare pairs of means. Statistically significant differences were determined at a 5% level of probability for all comparisons.

### Accession numbers

Sequence data from this study can be found in the GenBank data library under the following accession numbers: CsAPS (Csa11g074430, Csa18g014380 and Csa20g041200; accessions XM_010443947; XM_010483774 and XM_010495341, respectively), CsACT2 (XM_010489499, XM_010467690, XM_010508838), CsACT7 (XM_010424636, XM_010454680, XM_010493322), CsFAD3 (XM_010512122, XM_010471606, XM_010416024), CsFAE1 (XM_010439302, XM_101439301, XM_101434102), CsDGAT1 (XM_010417066, XM_010491178, XM_019236850), CsCRU1 (XM_010443649 and XM_010483472), CsSESA2 (XM_010449868, XM_010440350, XM_010449867), CsGWD1 (XM_010477668, XM_010460135, XM_010493565), CsISA3 (XM_010457014, XM_010437730, XM_010423523), CsBAM1 (XM_010490219, XM_010468522, XM_010515069), CsSUS1 (XM_010422626, XM_010456089, XM_010494893), CsGBS1 (XM_010480408, XM_010462780, XM_010503436), CsSBE2 (XM_010425379, XM_010492554, XM_010453956), CsSS1 (XM_010495271, XM_010456432, XM_010422950), CsSUT1 (XM_010479231 and XM_010461731), CsGLT1 (XM_010455468 and XM_010494194), CsMEX1 (XM_010494406, XM_010455648, XM_010425838), and CsTPT1 (XM_010483195, XM_010496474, XM_010443325).

## Additional file

**Additional file 1.** Additional figures and tables.
