# Supplementary material for: Seed-specific suppression of ADP-glucose pyrophosphorylase in Camelina sativa increases seed size and weight
Source: Biotechnol Biofuels. 2018 Dec 18;11:330. doi: 10.1186/s13068-018-1334-2 (PMC6297958; doi:10.1186/s13068-018-1334-2)
Supplement: Supplementary file 1 — Additional file 1. Additional figures and tables. [file 13068_2018_1334_MOESM1_ESM.docx]

**Additional file 1: Fig. S1.** Sequence comparison between AGPase small subunit genes (*APS*) in *Arabidopsis* *thaliana* and *Camelina sativa*. Primers for amplifying the RNAi fragment are underlined. Stars indicate identical nucleotides. Highlighted nucleotides show differences among Camelina *APS* genes.

At5g48300 ATGGCGTCTGTATCTGCAATTGGAGTTCTCAAGGTACCTCCT------GCTTCGACTTCC

Csa18g014380.1 ATGGCGTATGTCGCTGTAACTGGGGTTCTTAAGGTGCCTTCT------GCTTCGAGTTTC

Csa11g074430.1 ATGGCGTATCTCGCTGTAACTGGGGTTCTTAAGGTACCTGCTGCTTCTGCTTCGAGTTTC

Csa20g041200.1 ATGGCGTATGTCGCTGTAACTGGGGTTCTAAAGGTACCTTCTGCTTCTGCTTCGAGTTTC

*******.* *. *** ** ***.***** *****.*** ** ******* ** *

At5g48300 AATTCCACCG---GAAAAG---CCACGGAGGCGGTTCCCACGAG---GACTCTTTCTTTC

Csa18g014380.1 CATTCCACGGGCAGCAAAT---CCACTGAGGCGGTTCCGACGAGGAGTACTCTTTCTTTC

Csa11g074430.1 CATTCCACCGGCAGCAAAT---CCACTGAGGCGGTTCCGACGAGGAGTACTCTTTCTTTC

Csa20g041200.1 CATTCCACCGGCACCAAATCATCCACTGAGGCGGTTCCGACGAGGAGTACTCTTTCTTTC

.******* * .*** **** *********** ***** ************

At5g48300 TCCTCCTCTGTTACTTCATCCGACGACAAGATTTCACTCAAATCCACCGTCTCCCGTC--

Csa18g014380.1 TCCTCCTCT---------CTCGACGAGAATGTTTCACTCAGATCCACCGTCTCCCG----

Csa11g074430.1 TCCTCCTCT---------CTCGACGAGAATGTTTCACTCAGAGCCTCCGTCTCCCGTT--

Csa20g041200.1 TCCTCCTCT---------GTCGACGAGAATGATTCACTCAGAGCCATCGTCTCCCGTCGC

********* ****** ** .:********.* **: *********

At5g48300 -TTTGTAAATCTGTTGTTCGCAGGAATCCGATCATCGTCTCTCCCAAAGCCGTCTCAGAT

Csa18g014380.1 --TTGTGTCGGCCGAGATAGCAGGAATCCGATGATCGTCTCTCCTAAAGCCGTCTCCGAT

Csa11g074430.1 ----GTGTCGGCCGAGAGAGCAGGAATCCGATGATCGTCTCTCCTAAAGCCGTCTCCGAT

Csa20g041200.1 TTCTGCGTCGGCCGAGAGAGCAGGAATCCGATGATCGTGTCTCCTAAAGCCGTCTCCGAT

* .:. :*: .************* ***** ***** ***********.***

At5g48300 TCTCAAAACTCTCAAACTTGTCTCGATCCTGATGCTAGCAGCAGTGTTTTGGGGATAATC

Csa18g014380.1 TCTCAAAACTCACAAACTTGTCTCGATCCTGACGCCAGCAGCAGTGTTTTGGGGATTATC

Csa11g074430.1 TCTCAGAACTCACAAACTTGTCTCGATCCTGACGCCAGCAGCAGTGTTTTGGGGATTATC

Csa20g041200.1 TCTCAGAACTCACAAACTTGTCTCGATCCTGACGCCAGCAGCAGTGTTTTGGGGATTATC

*****.*****:******************** ** ********************:***

At5g48300 TTAGGAGGTGGAGCTGGAACTCGTCTTTATCCACTTACGAAGAAGAGAGCGAAACCAGCT

Csa18g014380.1 TTAGGAGGTGGAGCTGGGACTCGTCTTTATCCTCTTACCAAGAAGAGAGCGAAGCCAGCT

Csa11g074430.1 TTAGGAGGTGGAGCTGGGACTCGTCTTTATCCTCTCACCAAGAAGAGAGCGAAGCCAGCT

Csa20g041200.1 TTAGGAGGTGGAGCTGGGACTCGTCTTTATCCTCTTACCAAGAAGAGAGCGAAGCCAGCT

*****************.**************:** ** **************.******

At5g48300 GTGCCTCTTGGTGCTAACTATAGGCTTATTGATATTCCTGTGAGCAACTGTTTGAATAGC

Csa18g014380.1 GTTCCCCTTGGTGCTAACTATAGGCTTATTGATATCCCTGTTAGCAACTGTTTGAATAGC

Csa11g074430.1 GTTCCCCTTGGTGCTAACTATAGGCTTATTGATATCCCTGTTAGCAACTGTTTGAATAGC

Csa20g041200.1 GTTCCCCTTGGTGCTAACTATAGGCTTATTGATATCCCTGTTAGCAACTGTTTGAATAGC

** ** ***************************** ***** ******************

At5g48300 AACATATCCAAGATCTATGTTCTTACTCAGTTCAATTCCGCGTCTTTGAATCGTCATCTT

Csa18g014380.1 AACATCTCCAAGATCTATGTCCTTACTCAGTTCAATTCCGCCTCTCTCAATCGTCATCTC

Csa11g074430.1 AACATCTCCAAGATCTATGTCCTTACTCAGTTCAATTCCGCCTCTCTCAATCGTCATCTC

Csa20g041200.1 AACATCTCCAAGATCTATGTCCTTACTCAGTTCAATTCCGCCTCTCTCAATCGTCATCTC

*****.************** ******************** *** * ***********

At5g48300 TCACGAGCTTATGCTAGTAACATGGGAGGTTATAAGAATGAAGGATTCGTTGAAGTTCTC

Csa18g014380.1 TCTCGAGCTTATGCGAGTAACATGGGAGGTTACAAGAATGAAGGTTTTGTTGAAGTTCTT

Csa11g074430.1 TCTCGAGCTTATGCGAGTAACATGGGAGGTTACAAGAATGAAGGTTTTGTTGAAGTTCTT

Csa20g041200.1 TCTCGAGCTTATGCCAGTAACATGGGAGGTTACAAGAATGAAGGTTTTGTTGAAGTTC**TT**

**:*********** ***************** ***********:** ***********

At5g48300 GCTGCTCAACAGAGTCCTGAAAACCCCAACTGGTTCCAGGGGACAGCTGATGCCGTCAGG

Csa18g014380.1 GCTGCTCAACAAAGTCCTGAAAACCCCAACTGGTTCCAGGGGACAGCTGATGCCGTGAGG

Csa11g074430.1 GCTGCTCAACAAAGTCCTGAAAACCCCAACTGGTTCCAGGGGACCGCTGATGCGGTGAGG

Csa20g041200.1 **GCTGCTCAACAAAGTCC**TGAAAACCCCAACTGGTTCCAGGGGACAGCTGATGCCGTGAGG

***********.********************************.******** ** ***

At5g48300 CAATACTTGTGGTTGTTCGAGGAGCATAATGTCTTGGAGTATCTCATTCTTGCTGGGGAT

Csa18g014380.1 CAATACTTGTGGTTGTTTGAGGAGCATAATGTTTTGGAATATCTGATTCTTGCTGGGGAT

Csa11g074430.1 CAATACTTGTGGTTGTTTGAGGAGCATAATGTTTTGGAATATCTGATTCTTGCTGGGGAT

Csa20g041200.1 CAATACTTGTGGTTGTTTGAGGAGCATAATGTTTTGGAATATCTGATTCTTGCTGGGGAT

***************** ************** *****.***** ***************

At5g48300 CATTTGTATAGAATGGACTATGAGAAGTTTATTCAAGCACATAGGGAGACTGATGCTGAT

Csa18g014380.1 CATTTGTATCGAATGGATTACGAGAAGTTTATTCAAGCACATAGGGAGACTGATGCTGAT

Csa11g074430.1 CATTTGTATCGAATGGATTACGAGAAGTTTATTCAAGCACATAGGGAGACTGATGCTGAT

Csa20g041200.1 CATTTGTATCGAATGGATTACGAGAAGTTTATTCAAGCACATAGGGAGACTGATGCTGAT

*********.******* ** ***************************************

At5g48300 ATCACAGTAGCTGCATTACCAATGGACGAGCAACGAGCCACTGCTTTTGGGCTGATGAAG

Csa18g014380.1 ATCACAGTTGCTGCATTACCAATGGATGAGCAACGAGCCACTGCTTTTGGCCTGATGAAG

Csa11g074430.1 ATCACAGTTGCTGCATTACCAATGGATGAGCAACGAGCCACTGCTTTTGGCCTGATGAAG

Csa20g041200.1 ATCACTGTAGCTGCATTACCTATGGATGAGCAACGAGCCACTGCTTTTGGCCTGATGAAG

*****:**:***********:***** *********************** *********

At5g48300 ATTGATGAGGAAGGACGTATTATTGAATTTGCTGAAAAACCAAAAGGGGAGCACCTAAAG

Csa18g014380.1 ATTGATGAGGAAGGACGTATTGTTGAATTTTCCGAGAAACCAAAAGGGGAGCAACTTAAG

Csa11g074430.1 ATTGATGAGGAAGGACGTATTGTTGAATTTTCCGAGAAACCAAAAGGGGAGCAACTTAAG

Csa20g041200.1 ATTGATGAGGAAGGACGTATTGTTGAATTTTCTGAGAAACCAAAAGGGGAGCAGCTAAAG

*********************.******** * **.***************** **:***

At5g48300 GCCATGAAGGTTGACACAACAATTCTAGGTCTTGATGATCAGAGAGCCAAGGAGATGCCT

Csa18g014380.1 GCCATGAAGGTTGACACAACGATTCTAGGTCTTGACGATAAGAGAGCCAAGGAGATGCCT

Csa11g074430.1 GCCATGAAGGTTGACACAACGATTCTAGGTCTTGACGATAAGAGAGCCAAGGAGATGCCT

Csa20g041200.1 GCCATGAAGGTTGACACAACGATTCTAGGTCTTGACGATAAGAGAGCCAAGGAGATGCCT

********************.************** ***.********************

At5g48300 TTCATTGCTAGTATGGGTATTTATGTTGTAAGCAGAGATGTAATGCTAGACTTACTACGG

Csa18g014380.1 TACATTGCTAGTATGGGTATTTATGTTGTTAGCAGAGATGTAATGCTCGAGTTACTACGC

Csa11g074430.1 TACATTGCTAGTATGGGTATTTATGTTGTTAGCAGAGATGTAATGCTCGAGTTACTACGC

Csa20g041200.1 TACATTGCCAGTATGGGTATTTATGTTGTAAGCAGAGATGTAATGCTCGAGTTACTACGC

*:****** ********************:*****************.** ********

At5g48300 AATCAGTTTCCTGGAGCTAATGACTTTGGAAGTGAAGTCATTCCCGGTGCCACTTCCCTT

Csa18g014380.1 AACAAGTTTCCTGGAGCTAATGACTTTGGAAGTGAAGTCATTCCCGGTGCCACTGACCTT

Csa11g074430.1 AACAAGTTTCCTGGAGCTAATGACTTTGGAAGTGAAGTCATTCCCGGTGCCACTGACCTT

Csa20g041200.1 AACAAGTTCCCTGGAGCTAATGACTTTGGAAGTGAAGTCATTCCCGGTGCCACTGACCTT

** .**** ********************************************* .****

At5g48300 GGACTGAGGGTGCAAGCTTACCTATATGATGGTTACTGGGAAGACATTGGTACTATAGAG

Csa18g014380.1 GGACTGAGAGTGCAAGCTTACCTATATGATGGATACTGGGAAGACATCGGTACTATAGAG

Csa11g074430.1 GGACTGAGAGTGCAAGCTTACCTATATGATGGATACTGGGAAGACATCGGTACTATAGAG

Csa20g041200.1 GGACTGAGAGTGCAAGCTTACCTATATGATGGATACTGGGAAGACATCGGTACTATAGAG

********.***********************:************** ************

At5g48300 GCATTTTATAACGCTAATCTTGGAATCACCAAGAAACCAGTTCCTGATTTTAGTTTCTAT

Csa18g014380.1 GCATTCTATAACGCTAATCTCGGAATCACCAAGAAACCAGTTCCTGATTTTAGTTTCTAT

Csa11g074430.1 GCATTCTATAACGCTAATCTCGGAATCACCAAGAAACCAGTTCCTGATTTTAGTTTCTAT

Csa20g041200.1 GCATTCTATAACGCTAATCTCGGAAT**CACCAAGAAACCAGTTCC**TGATTTTAGTTTCTAT

***** ************** ***************************************

At5g48300 GACCGTTCTGCTCCGATCTACACACAGCCGCGTTATTTACCACCGTCTAAGATGCTTGAT

Csa18g014380.1 GATCGTTCCGCTCCCATCTATACACAGCCACGTTATTTACCACCGTCTAAGATGCTTGAT

Csa11g074430.1 GATCGTTCCGCTCCCATCTATACACAGCCACGTTATTTACCACCGTCTAAGATGCTTGAT

Csa20g041200.1 GATCGTTCGGCTCCCATCTATACACAGCCACGTTATTTACCACCGTCTAAGATGCTTGAT

** ***** ***** ***** ********.******************************

At5g48300 GCTGATGTTACTGACAGTGTCATCGGAGAGGGCTGTGTTATCAAGAACTGCAAAATTCAT

Csa18g014380.1 GCTGATGTCACGGACAGTGTCATCGGAGAGGGCTGTGTTATCAAGAACTGCAAAATCCAT

Csa11g074430.1 GCTGATGTCACGGACAGTGTCATCGGAGAGGGCTGTGTTATCAAGAACTGCAAAATCCAT

Csa20g041200.1 GCTGATGTCACGGACAGTGTCATCGGAGAGGGCTGTGTTATTAAGAACTGCAAAATCCAT

******** ** ***************************** ************** ***

At5g48300 CACTCTGTGGTTGGACTCCGTTCCTGCATATCAGAAGGTGCTATTATTGAAGATTCGTTA

Csa18g014380.1 CACTCTGTGATTGGACTCCGTTCCTGCATATCAGAAGGTGCTATTATTGAAGATTCGTTA

Csa11g074430.1 CACTCCGTGATTGGACTCCGTTCCTGCATATCAGAAGGTGCTATTATTGAAGATACGTTA

Csa20g041200.1 CACTCCGTGATTGGACTCCGTTCCTGCATATCAGAAGGTGCTATTATTGAAGATACGTTA

***** ***.********************************************:*****

At5g48300 TTAATGGGAGCTGATTATTACGAGACTGCTACGGAAAAGAGCCTCTTAAGCGCGAAAGGA

Csa18g014380.1 TTAATGGGAGCTGACTATTACGAGACTGCTTCGGAAAAGAGCCTCCTAAGCGCCAAAGGA

Csa11g074430.1 TTAATGGGAGCTGACTATTACGAGACTGCTTCGGAAAAGAGCCTCCTAAACGCCAAAGGA

Csa20g041200.1 TTAATGGGAGCTGACTATTACGAGACTGCTTCGGAAAAGAGCCTCCTAAGCGCCAAAGGA

************** ***************:************** ***.*** ******

At5g48300 AGTGTACCCATAGGTATTGGGAAAAACTCGCACATCAAAAGGGCCATCATCGACAAAAAC

Csa18g014380.1 AGTGTACCCATAGGTATTGGGAAAAATTCTCACATCAAAAGGGCCATCATTGACAAAAAT

Csa11g074430.1 AGTGTACCCATAGGTATTGGGAAAAATTCTCACATCAAAAGGGCCATCATTGACAAAAAT

Csa20g041200.1 AGTGTACCAATAGGTATTGGGAAAAATGCTCACATCAAAAGGGCCATCATTGACAAAAAT

********.***************** * ******************** ********

At5g48300 GCACGTATCGGTGACAATGTCAAGATCATAAACAGCGACAACGTGCAAGAGGCAGCGAGA

Csa18g014380.1 GCACGTATTGGTGACAATGTCAAGATCATAAACAGCGATAACGTGCAAGAATCAGCAAGA

Csa11g074430.1 GCACGTATTGGTGACAATGTCAAGATCATAAACAGCGATAACGTGCAAGAGTCAGCAAGA

Csa20g041200.1 GCACGTATTGGTGACAATGTCAAGATCATAAACAGCGATAACGTGCAAGAGTCAGCGAGA

******** ***************************** ***********. ****.***

At5g48300 GAGACTGATGGATATTTCATAAAGAGCGGAATTGTAACGGTTATCAAAGACGCCTTAATC

Csa18g014380.1 GAGACTGAAGGATATTTCATAAAGAGCGGAATTGTAACCGTTATGAAGGACGCCTTAATC

Csa11g074430.1 GAGACTGAAGGATATTTCATAAAGAGCGGAATTGTAACCGTTATGAAGGACGCCTTAATC

Csa20g041200.1 GAGACTGAAGGATATTTCATAAAGAGCGGAATTGTAACCGTTATGAAGGACGCCTTAATC

********:***************************** ***** **.************

At5g48300 CCAACCGGCACTGTCATCTGA

Csa18g014380.1 CCAGCCGGCACTGTCATCTAA

Csa11g074430.1 CCAGCCGGCACTGTCATCTAA

Csa20g041200.1 CCAGCCGGCACTGTCATCTAA

***.***************.*

**Additional file 1: Fig. S2**. Comparison of pod sizes in Suneson and AGPase RNAi lines during seed development. (**A**) Photos of pods taken at different developmental stages; (**B**) Differential pod size increases during seed development between Suneson and RNAi lines.

**Additional file 1: Fig. S3**. Fatty acid (FA) composition of transgenic seeds with suppressed AGPase is similar to the FA composition of wild type seeds grown in the field. Data represents average ± SE of six replicates. MT5=Suneson; AL2, 7, and 16 are transgenic RNAi lines.


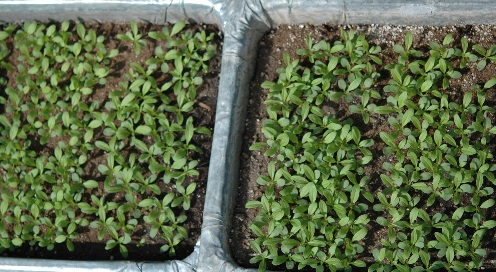

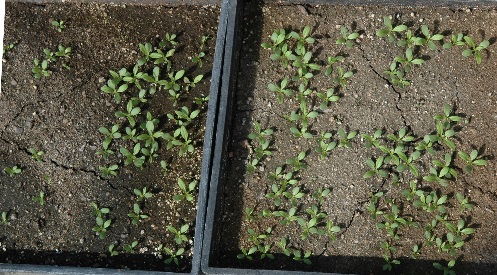

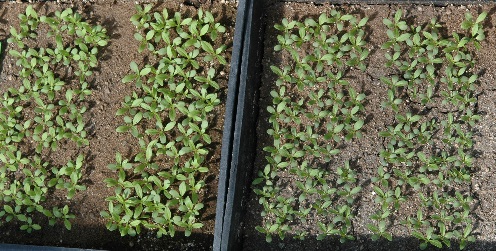


12.5 mm

25.0 mm

40 mm

A

B

C

**Additional file 1: Fig. S4**. Seed germination test in soil at different sowing depth in the greenhouse. (**A**) 12.5 mm, (**B**) 25 mm, (**C**) 40 mm.

Suneson AL2 AL7 AL16

**Additional file 1: Table S1**. List of primers used in construct

| Primer name | Sequence (5’ to 3’) | Restriction site |
| --- | --- | --- |
| AGPS1-F | GGATCCTCGCTGCTCAACAGAGTCC | BamHI |
| AGPS1-R | GCTAGCAGGAACTGGTTTCTTGGTG | NheI |
| AGPS1AS-F | CTCGAGTCGCTGCTCAACAGAGTCC | XhoI |
| AGPS1AS-R | CTGCAGAGGAACTGGTTTCTTGGTG | PstI |
| FAD2 intron-F | GCTAGCGTAAGGAAATAATTATTTTC | NheI |
| FAD2 intron-R | CTGCAGCTGTAATCAATCCAAATGTA | PstI |
| AGPase-RT-F | AATGACAGTGCCGGCTGGGA |  |
| AGPase-RT-R | CAGCTGTTCCCCTTGGTGCT |  |

**Additional file 1: Table S2**. List of primers used in semi-qRT-PCR

| Primer name | Sequence (5’ to 3’) | Primer name | Sequence (5’ to 3’) |
| --- | --- | --- | --- |
| CsACT7-For | CTATTCCAGCCATCGCTCAT | CsBAM1-For | GAAAGCACGGGTGTCAAAAT |
| CsACT7-Rev | GATTGATCCTCCGATCCAGA | CsBAM1-Rev | GCGTTTTCACCAGCTAGAGG |
| CsACT2-For | AGCAGGAGATGGAGACCTCA | CsSUS1-For | GGGAGGAGACAGGAGGAAAG |
| CsACT2-Rev | CTTGGTGCAAGTGCTGTGAT | CsSUS1-Rev | TCCACAACAGTCAACCCAAA |
| CsFAD3-For | CCGCCGTGTATTTTAACAGCT | CsGBS1-For | GAAGCTCTTCAGGCTGCTGT |
| CsFAD3-Rev | CGTCCAACCACATCACAAAGATG | CsGBS1-Rev | GCTGGGGACAATGATGAAGT |
| CsFAE1-For | CTGCACCGAGAACTTTCATCAAC | CsSBE2-For | CTTTCTGATGGGAGCGTGAT |
| CsFAE1-Rev | CCCTCGAACCTCTTCAGGAAT | CsSBE2-Rev | CTGCTGGTCCAGTGGAAGTT |
| CsDGAT1-For | TGCTTCTTCCACCTTTGGTT | CsSS1-For | ACGCAATGAGATACGGAACC |
| CsDGAT1-Rev | TTGAGCCGAACCTTTCTTGT | CsSS1-Rev | GCAGCGTTTTCCCAAGAATA |
| CsCRU1-For | TTCGGACCTGAGGTTATTGC | CsSUT1-For | CCCAAGCCATTACGTTCAGT |
| CsCRU1-Rev | GAGACGGGTCATCGAGGTTA | CsSUT1-Rev | TAACGCCAATATGCCACTCA |
| CsSESA2-For | GCAAACAAGCTCTTCCTCGT | CsGLT1-For | TGGACAAAATGGGAAGGAAA |
| CsSESA2-Rev | GTGGGTTCTCCATGTCGTCT | CsGLT1-Rev | GGGATGCGAATATCTCTGGA |
| CsGWD1-For | GAACAGAGATGGGAGCAAGC | CsMEX1-For | TGTTCATGTGGATGCCAGTT |
| CsGWD1-Rev | CCAGAAGATGGGTTCGTTGT | CsMEX1-Rev | ACCGCAAAGAATGACTTGCT |
| CsISA3-For | TCCTAGCTTTCACGCTCCAT | CsTPT1-For | TCTTCGTCTGCATTCCTCCT |
| CsISA3-Rev | TGGGGCCACATTGTAGGTAT | CsTPT1-Rev | TTTCCAACAGCGTGAGTCAG |

**Additional file 1: Table S3**. Summary of characteristics of wild type and AGPase suppression transgenic lines grown in field

| Traits | Year | Suneson | AL2 | AL7 | AL16 |
| --- | --- | --- | --- | --- | --- |
|  |  |  |  |  |  |
| Height (cm) | 2014 | 83.5±2.9 | 83.1±1.6 | 86.5±4.2 | 86.0±2.6 |
|  | 2015 | 66.4±4.4 | 66.3±5.4 | 60.2±2.6 | 63.9±3.4 |
|  | 2016 | 62.5±4.8 | 70.9±8.5 | 70.5±10.0 | 65.2±8.5 |
| Seeds/plant | 2015 | 921±208.7 | 987±108.4 | 941±409.1 | 1188±262.8 |
|  | 2016 | 5372.0±2039.0 | 3537.5±1332.2 | 3411.4±1592.8 | 4390.8±1659.7 |
| Seeds/pod | 2015 | 12.8±0.8 | 12.2±1.0 | 12.6±1.0 | 13.4±1.2 |
|  | 2016 | 12.6±0.7 | 13.2±0.6 | 13.1±0.8 | 12.8±0.7 |
| Yield (g) /plant | 2015 | 10.1±3.3 | 16.0±6.7 | 12.1±4.6 | 13.8±5.2 |
|  | 2016 | 15.0±4.4 | 17.3±4.1 | 18.5±6.1 | 21.2±5.8 |
| 100 seed weight (mg) | 2014 | 102.3±11.8 | 128.5±10.6 | 127.5±8.1 | 135.1±4.4 |
|  | 2015 | 94.8±10.3 | 138.4±5.1 | 146.1±4.7 | 143.9±9.6 |
|  | 2016 | 88.9±7.5 | 128.5±6.0 | 130.4±8.1 | 123.6±5.0 |
| Oil % | 2014 | 36.8±1.1 | 34.0±1.2 | 33.9±0.9 | 35.9±0.7 |
|  | 2015 | 37.0±1.8 | 38.8±0.6 | 38.6±1.1 | 38.6±0.6 |
|  | 2016 | 33.2±1.2 | 33.5±1.3 | 31.2±0.9 | 33.5±0.4 |
